# Vitiligo-Associated Autoimmune Disorders: A Woman With Vitiligo and Incipient Hypothyroidism

**DOI:** 10.7759/cureus.19164

**Published:** 2021-10-31

**Authors:** Sara Malik, Philip R Cohen

**Affiliations:** 1 Dermatology, Northwestern University, Feinberg School of Medicine, Chicago, USA; 2 Dermatology, University of California, Davis Medical Center, Sacramento, USA

**Keywords:** vitiligo, triiodothyronine, thyroxine, thyroid, stimulating, melanocyte, hypothyroidism, hormone, autoimmune, antibodies

## Abstract

Vitiligo is a skin condition that causes loss of pigmentation, resulting in hypopigmented and depigmented patches on the skin. Vitiligo has been associated with many autoimmune conditions. A 27-year-old female with a history of vitiligo had a clinical presentation and laboratory studies that were consistent with incipient hypothyroidism. The relationship between vitiligo, hypothyroidism, and other autoimmune conditions is discussed.

## Introduction

Vitiligo is an autoimmune disease characterized by hypopigmented and depigmented patches on the skin. Examination of the affected skin shows diminished pigment-producing melanocytes. Some of the treatments for vitiligo include topical corticosteroids, topical calcineurin inhibitors, and phototherapy. Recent advances in Janus kinase (JAK) inhibitors are promising for patients with extensive vitiligo; indeed, topical ruxolitinib has shown to be effective in treating vitiligo [1,2].

Thyroid disease occurs with either abnormally elevated or decreased amounts of thyroid hormone. Hyperthyroidism is generally characterized by excess thyroid hormone with decreased serum thyroid-stimulating hormone and elevated triiodothyronine and thyroxine levels. Patients may present with symptoms such as anxiety and tachycardia [3]. In contrast, hypothyroidism is generally characterized by decreased thyroid hormone synthesis and elevated thyroid-stimulating hormone, and low triiodothyronine and thyroxine levels, leading to symptoms such as cold intolerance, constipation, dry skin, myalgias, and vocal changes [4].

A 27-year-old female with onset of vitiligo six years ago presented for evaluation. During the prior six months, she was experiencing cold intolerance, constipation, and fatigue. Laboratory studies showed elevated thyroid peroxidase antibodies and thyroid-stimulating hormone, consistent with incipient hypothyroidism. The relationship between vitiligo and autoimmune diseases is discussed.

## Case presentation

A 27-year-old female with polycystic ovarian syndrome, acne (which was being treated topically with clindamycin 1% solution twice daily and tretinoin 0.025% cream each evening), and vitiligo presented for evaluation. She was not on any systemic medications and had been experiencing cold intolerance, constipation, and fatigue for the last six months. She was diagnosed with polycystic ovarian syndrome at the age of 16 years. At the age of 21 years, she observed vitiligo on her right thigh (after removal of a benign nevus at the site). Subsequently, she developed depigmentation in the vaginal area, axilla, right wrist, and right upper eyelid; during the prior year, the number and size of the lesions were increased. The patient’s family history was significant for her mother having diabetes and her father having thyroid cancer.

**Figure 1:**
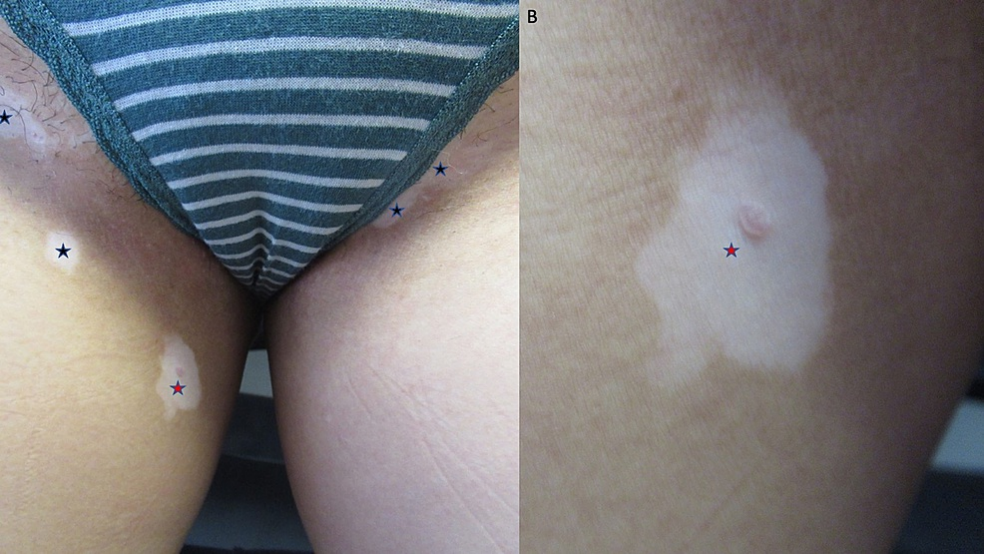
Vitiligo on the right and left thigh Distant (A) and closer (B) views showing hypopigmentation and depigmentation on the right and left proximal thighs of a 27-year-old female (black stars). The initial site of vitiligo (red star) on her right thigh occurred following the removal of a benign pigmented lesion at the site.

**Figure 2:**
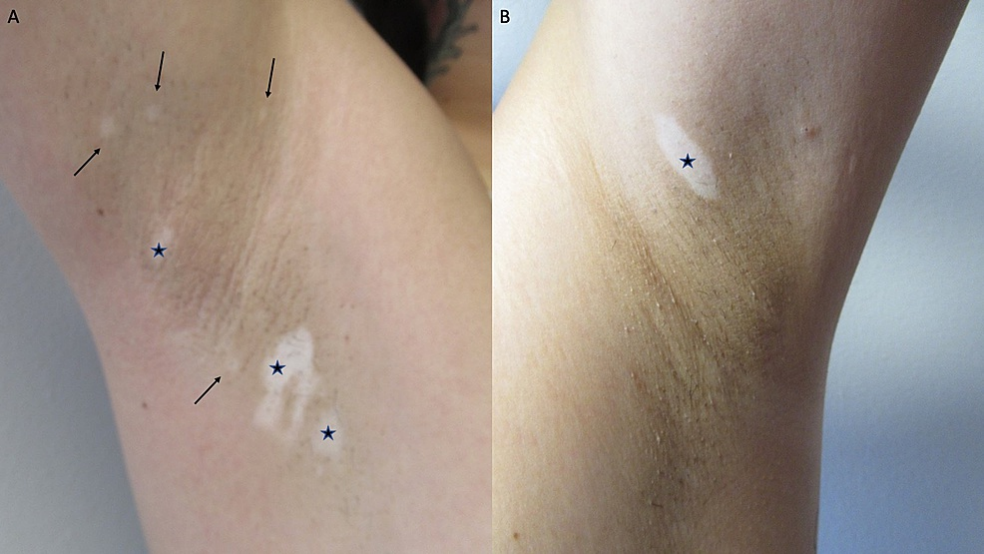
Hypopigmentation and depigmentation on the right and left axilla Vitiligo affecting the right (A) and left (B) axilla. Larger (black stars) and smaller (black arrows) hypopigmented and depigmented lesions were observed.

**Figure 3:**
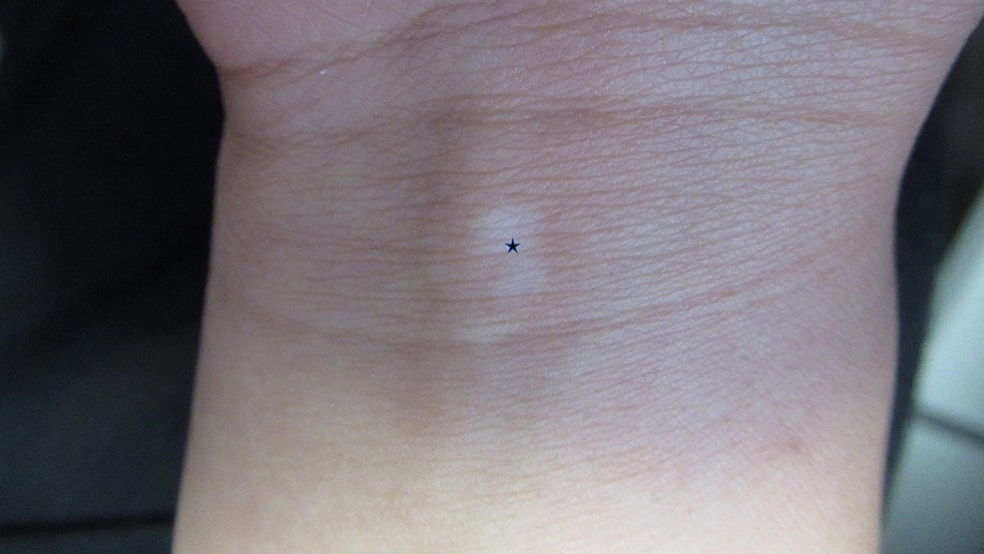
Vitiligo on the right flexor wrist A depigmented patch (black star) on the right flexor wrist of a 27-year-old female; laboratory evaluation demonstrated increased thyroid-stimulating hormone and markedly elevated thyroid peroxidase antibody consisted with incipient hypothyroidism.

**Figure 4:**
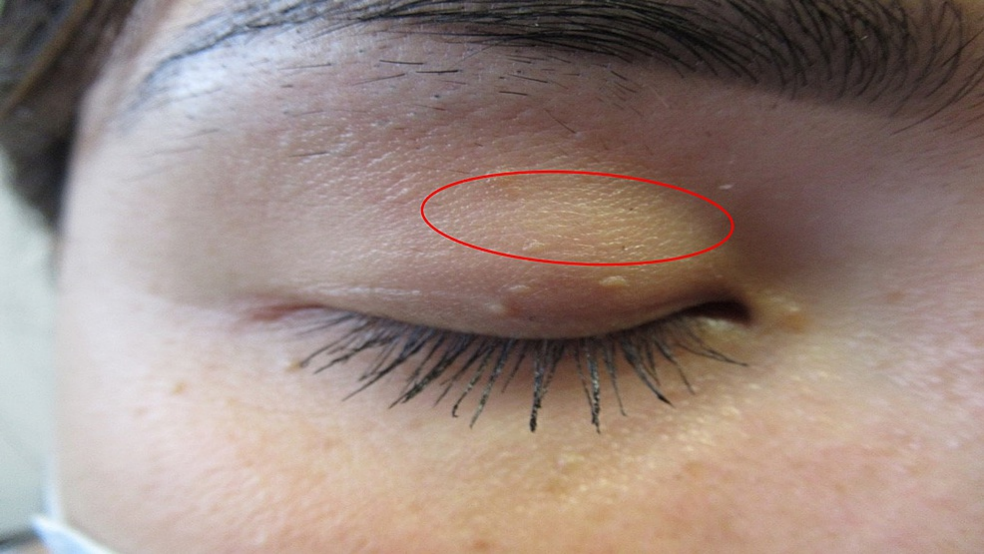
Depigmented patch on the right upper eyelid The depigmented patch of vitiligo (red circle) is located on the right upper eyelid of a 27-year-old female.

Cutaneous examination showed hypopigmented and depigmented patches on her thighs bilaterally (Figure 1), vulva, bilateral axilla (Figure 2), right flexor wrist (Figure 3), and right upper eyelid (Figure 4).

Laboratory studies showed elevated thyroid peroxidase antibodies (211 IU/mL; normal: <9 IU/mL) and elevated thyroid-stimulating hormone (6.7 mIU/L; normal: <4.5 mIU/L). She had a low antinuclear antibody titer with a nuclear, dense, and fine speckled pattern; however, all her other lupus serologies (such as anti-double-stranded DNA, anti-ribonucleoprotein, anti-scleroderma-70, anti-Sjogren’s syndrome-related antigen A, anti-Sjogren’s syndrome-related antigen B, and anti-Smith antibody) were negative. Her triiodothyronine (120 ng/dL; normal: 76-181 ng/dL) and thyroxine (5.6 mcg/dL; normal: 5.1-11.9 mcg/dL) levels were normal. Her white blood cell count (3600/uL; normal: 3800-10,800/uL) and absolute neutrophils (1346/uL; normal: 1500-7800 cells/uL) were below normal; the remainder of her complete blood count and serum chemistries were normal. Her glucose was normal (82 mg/dL; 65-99 mg/dL). Additional antibody tests (such as anti-glutamic acid decarboxylase 65 and islet-antigen 2) were not performed. 

The correlation between her clinical presentation and the laboratory studies established the diagnosis of vitiligo associated with incipient hypothyroidism. The initial management of the vitiligo was topical fluocinonide 0.05% cream applied twice daily to the areas of decreased or absent pigment. She was also referred to an endocrinologist for consideration of treatment with thyroid supplementation; additional assessment for other endocrinology-associated syndromes (such as polyglandular autoimmune syndromes) may also have been considered.

## Discussion

The pathogenesis of vitiligo remains to be definitively established, and several mechanisms have been postulated. The adhesion defect theory suggests that trauma can result in the detachment of melanocytes. The biochemical theory postulates that changes to the redox balance can destroy melanocytes. The neuronal theory hypothesizes that epidermal nerve endings release catecholamine, which is cytotoxic to melanocytes. The autoimmune theory proposes that patients with vitiligo have autoimmunity in which there is a destruction of melanocytes as a result of genetic and environmental factors. Indeed, vitiligo has also developed concurrently in patients with other autoimmune conditions, such as thyroid disease [2,5,6].

In patients with suspected thyroid disease, laboratory evaluation should include thyroid-stimulating hormone, triiodothyronine, and thyroxine. In addition, the evaluation of thyroid-associated antibodies should be considered, such as thyroid peroxidase antibody (previously referred to as microsomal antigen) and thyroglobulin antibody. Thyroid peroxidase is a protein that iodinates tyrosine residues in the thyroglobulin molecule. Anti-thyroid peroxidase antibodies are involved in the destruction of thyrocytes, which are responsible for the production of thyroid hormones. Thus, most patients with thyroid disease will test positive for thyroid peroxidase antibody [7,8].

Patients with thyroid disease can also test positive for thyroglobulin antibodies. Thyroglobulin is a protein made by the thyroid gland. In patients with thyroid disease, CD8+ T-cells recognize thyroglobulin and play a role in thyroid destruction [8,9].

Several studies have observed that patients with vitiligo have an increased prevalence of autoimmune thyroid disease. In a study of 94 patients with vitiligo and 96 patients in the control, anti-thyroid peroxidase levels were measured. A significant difference was noted in anti-thyroid peroxidase levels between patients with vitiligo and patients in the control group; thyroid peroxidase was found in 18% of the patients who had vitiligo compared with 7% of the patients in the control group (P-value < 0.025). The researchers, based on this observation, suggested that there may be a correlation between vitiligo and thyroid disease [7].

Furthermore, in a study of 33 patients with vitiligo, 27% of the participants had elevated anti-thyroglobulin antibody and 24% had elevated anti-thyroid peroxidase antibodies. In contrast, only one patient in the control group, consisting of patients without vitiligo, had abnormalities in thyroid hormonal status, and only two patients had positive thyroid autoantibodies. Additionally, patients with vitiligo had a significantly higher frequency of both anti-thyroglobulin and anti-thyroid peroxidase (P-value < 0.05) [10]. 

A cross-sectional study investigated thyroid dysfunction in patients with vitiligo; the study subjects consisted of 109 Iranian patients who were diagnosed with vitiligo at least six months before the start of the study. The results showed that 37% of patients were positive for anti-thyroid peroxidase antibody and 32% were positive for anti-thyroglobulin antibody. The investigators concluded that patients with vitiligo should be tested for thyroid dysfunction [11].

Another study in India consisted of 35 patients with vitiligo and 30 patients without vitiligo in the control group. Of the patients with vitiligo, 31% were positive for anti-thyroid peroxidase; only 10% of the patients in the control group were positive for the antibody. The researchers emphasized that the study results demonstrated that patients with vitiligo have an increased likelihood of testing positive for thyroid autoantibodies [12].

In addition to thyroid diseases, such as Hashimoto’s disease and Graves’ disease, vitiligo has been observed in patients with other autoimmune conditions, including dermatologic, endocrine, hemolytic, and neurologic conditions (Table 1) [5,13-16]. Endocrine disorders associated with vitiligo include Addison’s disease and diabetes. Vitiligo-associated dermatologic disorders include alopecia areata, atopic dermatitis, and psoriasis [15,16]. Vitiligo has also been observed in individuals with hematologic conditions such as idiopathic thrombocytopenic purpura and pernicious anemia. Also, vitiligo has also coexisted with neurologic conditions such as multiple sclerosis and myasthenia gravis [14-16].

**Table 1:** Autoimmune conditions associated with vitiligo

Condition	References
Dermatologic disorder	[15,16]
Alopecia areata	[15,16]
Atopic dermatitis	[15,16]
Psoriasis	[15,16]
Endocrine disorder	[13–16]
Addison’s disease	[13]
Diabetes mellitus (type I)	[15,16]
Graves’ disease	[14–16]
Hashimoto’s disease	[14–16]
Hematologic disorder	[14–16]
Idiopathic thrombocytopenic purpura	[14–16]
Pernicious anemia	[14–16]
Neurologic disorder	[14–16]
Multiple sclerosis	[14–16]
Myasthenia gravis	[14–16]
Rheumatologic disorder	[5,14–16]
Rheumatoid arthritis	[5,14–16]
Sjogren’s syndrome	[5,15,16]
Systemic lupus erythematous	[14–16]

Our patient also had polycystic ovarian syndrome. However, there are only rare descriptions of women with polycystic ovarian syndrome and vitiligo. Antioxidants, vitamin D supplements, and metformin were reported to have a positive effect on a 37-year-old patient with both polycystic ovarian syndrome and vitiligo, resulting in repigmentation on the face, weight reduction, and reduction in hypertrichosis. Another patient, a 24-year-old female with vitiligo who was undergoing laser hair removal for polycystic ovarian syndrome-associated hypertrichosis, was described [17,18].

Laboratory studies should be considered to evaluate a patient with vitiligo. They include lupus erythematosus serologies (antinuclear antibody titer, anti-double-stranded DNA antibody, anti-ribonucleoprotein antibody, anti-scleroderma-70 antibody, anti-Sjogren’s syndrome-related antigen A antibody, anti-Sjogren’s syndrome-related antigen B antibody, and anti-Smith antibody), complete blood cell and platelet counts, serum chemistries, and a thyroid function panel (thyroid-stimulating hormone, triiodothyronine, and thyroxine) [5]. Since thyroid disease is prevalent in patients with vitiligo, it is reasonable to not only order a thyroid function panel but also thyroid antibodies (anti-peroxidase antibody and anti-thyroglobulin antibody). In addition, if there is unexplained anemia, in particular with macrocytic indices, laboratory evaluation for pernicious anemia (vitamin B12, folate, and parietal cell antibodies) should be considered.

Our patient had vitiligo; however, the number and size of her lesions were recently noted to be increasing. Her laboratory evaluation demonstrated a low antinuclear antibody titer with a nuclear staining pattern that is commonly seen in normal individuals and rarely associated with systemic lupus erythematosus and systemic sclerosis. Her other laboratory studies were negative for these conditions. Additionally, her laboratory evaluation showed mild leukopenia and neutropenia of undetermined significance. However, the detection of increased thyroid-stimulating hormone and markedly elevated thyroid peroxidase antibody were consistent with Hashimoto’s thyroiditis. In addition to the laboratory studies, she had symptoms (such as cold intolerance, constipation, and fatigue) of hypothyroidism for the last six months. Her clinical and laboratory findings prompted us to refer her to an endocrinologist who would consider initiation of treatment for hypothyroidism.

## Conclusions

Vitiligo is an autoimmune condition that can occur with other autoimmune conditions. These include endocrine, dermatologic, hematologic, neurologic, and rheumatologic conditions. Patients presenting with the new onset of vitiligo or with rapid progression of previously stable disease should be considered for evaluation of thyroid function and antibodies. Management of detected incipient thyroid disease (such as our patient with incipient hypothyroidism) or fully developed thyroid disease should be considered for the initiation of appropriate thyroid therapy.
